# Recent advances in expanding the coverage of the lipidome

**DOI:** 10.1016/j.copbio.2016.11.008

**Published:** 2017-02

**Authors:** Sergey Tumanov, Jurre J Kamphorst

**Affiliations:** 1 Cancer Metabolism Research Unit, Cancer Research UK Beatson Institute, Garscube Estate, Switchback Road, Glasgow G61 1BD, UK; 2 Institute of Cancer Sciences, University of Glasgow, Garscube Estate, Switchback Road, Glasgow G61 1QH, UK

## Abstract

The lipidome comprises a large array of molecules with diverse physicochemical properties. Lipids are structural components of cells, act as a source of energy, and function as signaling mediators. Alterations in lipid metabolism are involved in the onset and progression of a variety of diseases, including metabolic syndrome and cancer. Because of this, interest in lipidomics, the comprehensive characterization of the lipidome by mass spectrometry, has intensified in recent years. However, obtaining a truly complete overview of all lipids in a sample has remained very challenging due to their enormous structural diversity. Here, we provide an overview of the collection of analytical approaches used to study various lipid classes, emphasizing innovations in sample preparation and liquid chromatography–mass spectrometry (LC–MS). Additionally, we provide practical suggestions for increasing the coverage of the lipidome.

## Highlights

• The extreme diversity of the lipidome poses a significant analytical challenge.
• A variety of mass spectrometry-based approaches exist for the analysis of lipids.
• Combining extraction and separation procedures maximizes coverage of the lipidome.

## Graphical abstract

**Figure:**
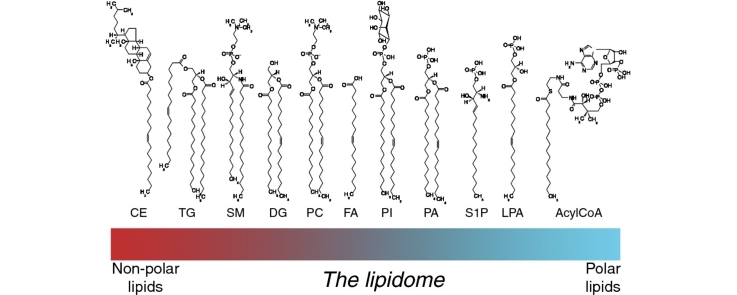

**Current Opinion in Biotechnology** 2017, **43**:127–133

This review comes from a themed issue on **Analytical biotechnology**

Edited by **Jurre J Kamphorst** and **Ian A Lewis**

For a complete overview see the Issue and the Editorial

Available online 1st December 2016

**http://dx.doi.org/10.1016/j.copbio.2016.11.008**

0958-1669/© 2016 The Author(s). Published by Elsevier Ltd. This is an open access article under the CC BY license (http://creativecommons.org/licenses/by/4.0/).

## Introduction

Lipids are low molecular weight biomolecules characterized by their high hydrophobicity. They are involved in nearly all major aspects of cell biology. For instance, triglycerides store energy in the form of fatty acids, phospholipids form cellular membranes, and various lipid classes initiate or transduce signaling events: lysophosphatidic acid stimulates proliferation and migration [1], and specific phosphatidylinositol lipid species transduce insulin signaling [2]. Because of their intricate involvement in many physiological processes, it is not surprising that lipids play important roles in a variety of diseases such as cancer, cardiovascular disorders, neurodegenerative diseases, obesity and diabetes [3, 4, 5, 6, 7].

Particularly due to their involvement in pathological processes, there is a strong interest in investigating the variety of lipids present in samples and their functional roles in disease. It has recently been estimated that mammalian cells contain 10 000 individual lipid species [8]. As much as 50% of these remain without assigned functions [9^••^]. Therefore, many novel structures with potential medical relevance are left to be discovered. This is perhaps best illustrated by the recent discovery of branched *f*atty *a*cid esters of *h*ydroxy *f*atty *a*cids (FAHFAs), which were found to improve glucose tolerance and to stimulate insulin secretion in diabetic mice [10^••^].

Lipidomics has emerged as a key technology for investigating the metabolism and cellular functions of known lipids, as well as for discovering and characterizing novel lipid structures. Lipidomics can be defined as the comprehensive characterization of lipids in biological systems [11]. In recent years, there have been considerable advancements in various aspects of the lipidomics ‘pipeline’. For instance, the generation of lipid databases and the tools to cross-compare them with experimentally obtained lipidomics data has been an important development. Examples are LipidMaps, LipidBank, LipidHome, LipidBlast, and LipidSearch [12•, 13, 14, 15, 16]. The lipidomics field has particularly benefited from continued developments in mass spectrometry; the ever-increasing sensitivity, resolution, speed, and dynamic range of modern instruments allow researchers to probe the lipid composition in unprecedented detail. These developments in mass spectrometry have been exploited in different ways. For example, direct infusion or ‘shotgun’ lipidomics approaches introduce samples into the mass spectrometer without prior separation, instead relying on the resolution and dynamic range of modern instruments [17, 18]. This approach enables the rapid analysis of samples, but is unable to resolve isobaric species and may compromise the detection of lower abundant species due to ion suppression effects and insufficient dynamic range. Instead, although requiring more time, liquid chromatography-based separation followed by mass spectrometry (LC–MS) remains very popular as a way to increase lipidome coverage, separate isobaric species and maximize dynamic range [17, 19, 20]. The enormous potential of LC–MS in comprehensive lipidomic analysis is arguably best demonstrated by recent study exploring the lipid composition of platelets, where approximately 5600 unique lipids were detected [9^••^].

It is important to note that, despite exciting advances in mass spectrometry and bioinformatics, the degree to which the lipidome can truly be ‘covered’ comprehensively actually depends on the sample extraction and liquid chromatography separation. Due to the considerable chemical diversity of lipids, any single extraction (and likewise separation) procedure will invariably create a bias toward certain lipid species at the expense of others. We therefore argue that combining multiple extraction and separation procedures is essential to maximize coverage of both the more hydrophilic and hydrophobic lipid classes. To support our argument, we provide an overview of recently published literature on lipid extraction and LC-MS procedures, and suggest a practical approach for maximizing the coverage of the lipidome.

## Lipid extraction

**Figure 1:**
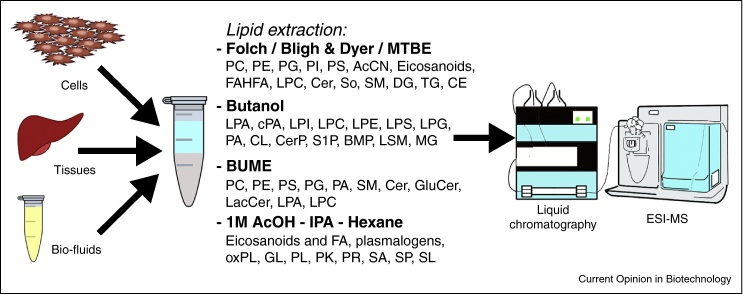
**Commonly used extraction procedures in lipidomics and the lipid classes they cover.** Published methods include chloroform and methyl *tert*-butyl ether (MTBE) based extractions [23, 24, 28, 29, 30, 31, 32], butanol and butanol-methanol (BUME) extraction procedures [17, 25, 33, 34, 35], and an extraction procedure using acetic acid (AcOH) with isopropanol and hexane [9^••^]. (L)PC, (lyso)phosphatidylcholine; (L)PE, (lyso)phosphatidylethanolamie; (L)PG, (lyso)phosphatidylglycerol; (L)PI, (lyso)phosphatidylinositol; (L)PS, (lyso)phosphatidylserine; AcCN, acyl-carnitine; FAHFA, branched fatty acid esters of hydroxy fatty acid; Cer, ceramide; So, sphingosine; (L)SM, (lyso)sphingomyelin; DG, diglyceride; TG, triglyceride; CE, cholesterol ester; (L)PA, (lyso)phosphatidic acid; cPA, cyclic phosphatidic acid; CL, cardiolipin; CerP, ceramide-phosphate; S(1)P, sphingosine-1-phosphate; BMP, bis(monoglyceride)phosphate; MG, monoglyceride; GluCer, glucosyl-ceramide; LacCer, lactosyl-ceramide; FA, fatty acid; oxPL, oxidized phospholipids; GL, glycerides; PL, phospholipids; PK, polyketides; PR, prenols; SL, sphingolipids.

Lipidomic sample preparation protocols exploit the hydrophobic nature of lipids to extract them while eliminating other components of the biological matrix (i.e. proteins, sugars, inorganic salts) that could potentially interfere with the chromatographic separation and mass spectrometry analysis (Figure 1) [21, 22]. As will be discussed below, the most commonly used lipid extraction procedures involve chloroform, as originally described by Folch et al. [23], and Bligh and Dyer [24]. More recently, extraction procedures with less hazardous solvents have been introduced [17]. Finally, several protocols for the extraction of more hydrophilic lipid species have been published [25, 26, 27].

### Chloroform-based lipid extraction

Variations on the lipid extraction protocol developed originally by Folch in 1957 remain very widely used to this day [23]. It efficiently extracts the abundant classes of lipids such as most phospholipids, glycerides, cholesterol and cholesterol-esters, sphingolipids, and waxes [28]. The original method is based on liquid-liquid partitioning using chloroform and methanol in a 2:1 (v/v) ratio [23]. A method modified to use less organic solvents and to be suitable for water-based samples was later described by Bligh and Dyer [24, 29]. Chloroform-based protocols directed toward specific lipid classes have also been published. For example, a method for extracting branched fatty acid esters of hydroxyl fatty acids (FAHFAs), a newly discovered class of endogenous mammalian lipids with antidiabetic and anti-inflammatory effects, uses a modified Bligh and Dyer extraction method from acidified serum samples [10^••^]. A downside of chloroform-based protocols is the inability to extract charged and more polar lipids like some of the lysophospholipids (LPA), phosphatidic acid lipids (PA), acylcarnitines, acyl-CoAs, and sphingosine phosphates [17].

### MTBE-based lipid extraction

Recently, extraction procedures using methyl *tert*-butyl ether (MTBE) have gained popularity as a less toxic alternative to chloroform [30]. An added advantage of MTBE is that in contrast to chloroform, its density is lower than water. Thus, MTBE will separate to the upper phase during extraction of water-based samples, facilitating a convenient removal of the lipid extract [31]. Importantly, it was also shown that a modified MTBE method can provide near quantitative recovery of more polar lipids such as LPAs and PAs [32]. A downside of using MTBE, however, is the significant carry-over of water, causing the typical sample drying step to be lengthy, and the increased chance for ion suppression and adduct formation due to the co-extraction of salts and other metabolites [17, 21].

### Butanol-based lipid extraction

An extraction procedure using butanol was originally used for the analysis of lysophospholipids and acylcarnitines [33]. Later, it was shown that butanol can additionally efficiently recover cardiolipins (CL), bis(monoacylglycero)phosphate (BMP), as well as their precursors phosphoglycerols (PGs) and phosphatidic acids (PAs) from heart tissues and primary human skin fibroblasts [25]. Because of this observation, this extraction procedure was modified for global lipid analysis [17]. In one procedure, mixing of a sample, first with butanol:methanol (3:1, v/v) leads to single extraction phase, or ‘BUME’ (BUtanol MEthanol) mixture. Thereafter, heptane:ethyl acetate (3:1, v/v) and 1% acetic acid are sequentially added to BUME mixture. The top organic layer of this extraction system contains most major lipid classes. This BUME method can be used as an alternative to MTBE with less salts and metabolites carrying over into the organic phase. Also, BUME extraction results in lipid recovery highly comparable to a modified Bligh-Dyer method [34, 35]. A consideration is, however, that co-extracted water in the organic phase may significantly increase the time needed for sample evaporation.

### Other solvent systems for lipid extraction

A few studies describe lipid extraction procedures employing alternative solvent systems such as methanol/dichloromethane (DCM), isopropanol, or a combination of acetic acid solution/isopropanol/hexane [9••, 36•]. The rationale for using DCM is that it is less toxic than chloroform, and extraction efficiency of plasma lipids appears comparable to chloroform [36^•^]. In this paper, a strong case is made for isopropanol as a precipitation solvent for plasma lipid analysis. In a study investigating the platelet lipidome, use of a hexane/isopropanol protocol with addition of 1 M acetic acid was found to be able to extract ∼5600 different lipid species including fatty acids, glycerolipids, phospholipids, polyketides, prenol lipids, saccharolipids, sphingolipids and sterol lipids [9^••^].

### Specialized lipid derivatization approaches for polar and low-abundant lipids

Many physiological processes in cells are regulated by low-abundant bioactive lipids, such as phosphoinositol phosphates, eicosanoids and prostaglandins [37, 38, 39, 40, 41]. Their analysis is easily compromised due to a combination of their low abundance, limited extraction efficiency in regularly used solvents, and susceptibility to ion suppression. Chemical derivatization of the polar head group of these lipids appears to be a promising approach to increase the stability, extraction efficiency, and ionization [40, 42, 43]. For example, it was demonstrated that phosphoinositol phosphates, mediators of insulin signaling and other signaling cascades, can be readily recovered, analyzed and quantified after methylation using a trimethylsilyl diazomethane solution [40]. Derivatization has also successfully been used for the analysis of fatty acids and eicosanoids [42, 43].

## Mass spectrometry-based lipidomics

Recent innovations in separation and analysis science have boosted lipidomics applications. Most lipidomics methods using mass spectrometry can be subdivided in two distinct strategies; those that directly infuse lipid extracts into the mass spectrometer, also called ‘shotgun’ lipidomics, and those where lipid samples undergo chromatographic separation prior to analysis, i.e. LC–MS.

### Direct infusion, or ‘shotgun’ lipidomics

Shotgun lipidomics is favored by many in the lipidomics community [44, 45, 46]. Here, lipid extracts are directly introduced into the mass spectrometer using electrospray ionization (ESI), often at the nano-scale [47, 48, 49]. This approach benefits significantly from high resolution instruments that have become increasingly available. One of the strong advantages of shotgun lipidomics is that sample analysis time can be on the order of minutes, making it amenable for high-throughput, routine analysis of major lipid species [21]. However, when the aim is to maximize coverage of the entire lipidome, several aspects need to be considered. First, the common occurrence of isobaric species complicates shotgun lipidomics approaches. For example, in our (LC–MS) lipidomic screens we frequently find phosphatidylcholine (PC) species that have the same exact mass as phosphatidylethanolamines (PEs). With LC–MS these species can be differentiated based on differences in retention time. This is not possible with shotgun lipidomics, and although fragmentation may provide a solution, its interpretation is not trivial [50]. Second, in-source fragmentation can easily confound lipid identification and quantification. From our own experience, lysophosphatidylcholines (LPCs) in negative mode can lose their choline moiety during ionization to become lysophosphatidic lipids (LPAs). Although this may only happen to a fraction of the LPCs, their massive over-abundance relative to LPAs can easily lead to significant over-estimation of LPA levels. Finally, as all lipids, both high and low abundant, are introduced into the mass spectrometer and analyzed simultaneously, ion-suppression and a limited dynamic range increase the likelihood of compounds in the lower concentration range being lost.

### Liquid chromatography–mass spectrometry

**Figure 2:**
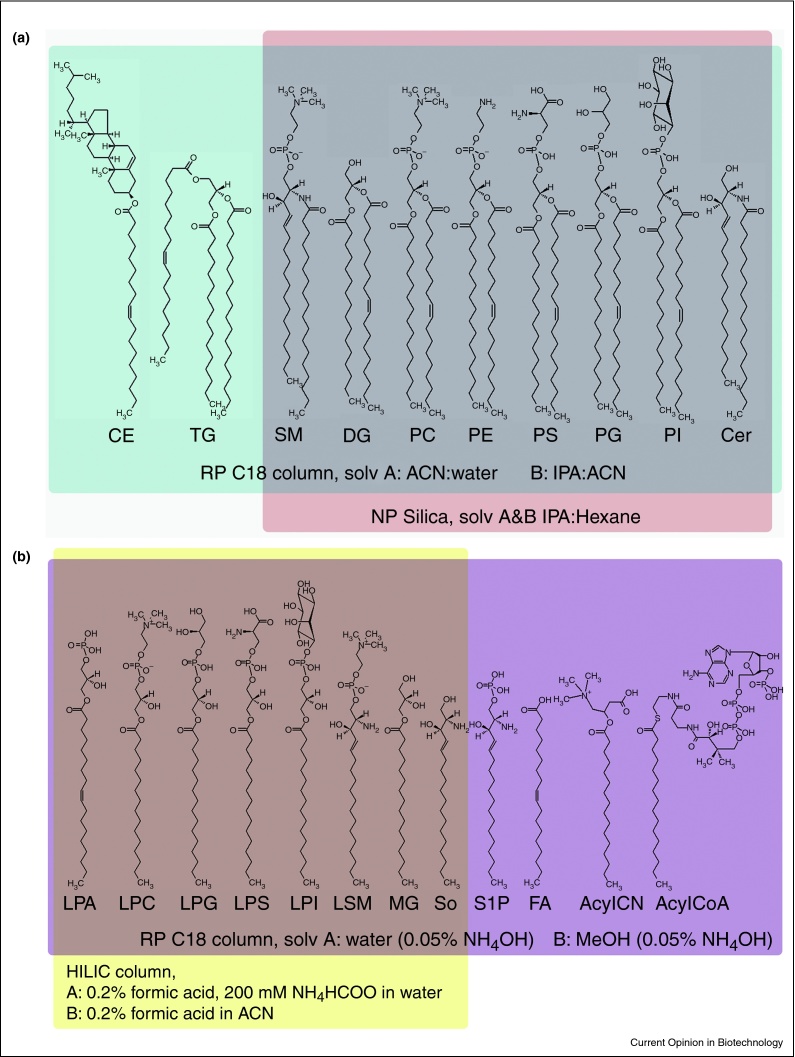
**Coverage of the (a) apolar, and (b) polar lipid classes by commonly used separation methods.** RP, reversed phase; ACN, acetonitrile; IPA, isopropanol; NP, normal phase; HILIC, hydrophilic interaction liquid chromatography; CE, cholesterol ester; TG, triglyceride; (L)SM, (lyso)sphingomyelin; DG, diglyceride; (L)PC, (lyso)phosphatidylcholine; PE, phosphatidylethanolamine; (L)PS, (lyso)phosphatidylserine; (L)PG, (lyso)phosphatidylglycerol; (L)PI, (lyso)phosphatidylinositol; Cer, ceramide; MG, monoglyceride; So, sphingosine; S1P, sphingosine-1-phosphate; FA, fatty acid; AcylCN, acyl-carnitine.

For the comprehensive characterization of the lipidome, liquid chromatography–mass spectrometry (LC–MS) remains the method of choice for many. At the cost of increased run times, LC separations reduce the complexity of the eluent introduced into the mass spectrometer. This reduces the risk for ion-suppression during electrospray ionization, and is less tasking for the mass spectrometer with respect to the dynamic range. As a result, low abundant (and often biologically relevant) species are more readily detected. Various modes of separation have been used for lipid analysis (Figure 2). For example, normal phase chromatography has been used in the past to separate phospholipid classes based on head group polarity [3, 51]. More recently, hydrophilic interaction liquid chromatography (HILIC) was used for the separation of lipid extracts [19, 52]. It is worth mentioning that HILIC-based separations appeared particularly suitable for separating lysophospholipid regioisomers (mono-acyl lipids with same fatty acid carbon length but a different double bond position) [19]. Reversed phase chromatography appears to be the most popular mode of separation. A very typical separation involves a C_8_/C_18_ column and a gradient of (mobile phase A) acetonitrile–water (60:40), and (mobile phase B) isopropanol–acetonitrile (90:10), with gradient run times commonly lasting 10–40 min depending on the flow rate used [53, 54]. This platform is very suitable for the analysis of many of the phospholipids species (PC, PE, PG, PI, PS), sphingomyelins, ceramides, cholesterol-esters, diglycerides, and triglycerides. Although lysophospholipids and phosphatidic acid (PA) lipids can also be detected with this setup, a more polar gradient, such as a methanol-water gradient, results in our experience in a better analytical performance, i.e. improved peak shape and ionization.

Two recent studies in particular demonstrate the merit of LC–MS-based lipidomics for the detection and characterization of unknown lipids. In one study lipidomic analysis was performed on the adipose tissue of both wildtype mice and mice with adipose tissue-specific overexpression of the glucose transporter Glut4, which leads to increased glucose tolerance [10^••^]. Tissue lipids were extracted using a modified Bligh and Dyer protocol and subsequently analyzed in both positive (on a C_5_ column) and negative (on C_18_ column) mode. In both cases a 45 min gradient of (A) 95:5 v/v water:methanol and (B) 60:35:5 v/v isopropanol:methanol:water was used, with in negative mode additionally 0.1% ammonium hydroxide and in positive mode 0.1% formic acid plus 5 mM ammonium formate. The mass spectrometer used was an Agilent 6220 TOF instrument. Untargeted data analysis was then performed using XCMS [55^•^], resulting in the identification of a cluster of novel lipids that were elevated in the Glut4 overexpressing mice. Follow up work revealed that these molecules are branched fatty acid esters of hydroxyl fatty acids (FAHFAs), which were found to have strong antidiabetic and anti-inflammatory effects. In the second study a very comprehensive analysis was conducted to map the lipidome remodeling upon platelet activation [9^••^]. Here, extraction was performed with a 2:20:30 v/v 1 M acetic acid:isopropanol:hexane mixture. Samples were then subjected to two gradient setups, one a 55 min 50:50 v/v acetonitrile:water and 70:30 v/v isopropanol:acetonitrile gradient for the analysis of the ‘non-polar’ lipids (lipophilic phospholipids, neutral lipids), and the other a 30 min gradient based on 75:25 v/v water:acetonitrile and 60:40 methanol:acetonitrile, for the analysis of ‘polar’ species (eicosanoids, fatty acids). The analysis was done on an Orbitrap elite in full scan only, and for both gradients samples were run both in positive and negative mode and at two mass ranges (100–900 and 900–1800 *m*/*z*), totaling 8 runs per condition. In this tour-de-force profiling study, data was then analyzed with a program called Sieve and the resulting list of lipid features was interrogated against multiple databases (HMDB, Lipidhome, LipidMaps, and METLIN). This led to the detection of approximately 5600 unique lipid species, of which only 50% could be putatively identified. Of these 5600 lipids, 900 increased upon platelet activation by thrombin.

## Conclusion and future perspectives

Cells, tissues, and bio-fluids contain many thousands of structurally diverse lipids. In one of the most comprehensive lipidomics screens thus far, only 50% of the ∼5600 detected lipids could be identified [9^••^]. While the numbers detected and percentages identified might differ from one biological system to the next, it is apparent that tremendous opportunities exist to discover new aspects of lipid biology with potential relevance to medicine. Lipidomics will continue to play a key role in seizing these opportunities.

We conclude that no single extraction and separation gradient modality sufficiently captures the full spectrum of chemical diversity that exists between lipid classes. Rather, a combination of sample extraction and separation procedures is required. For example, a good approach would be to subject samples to both a chloroform-based extraction in conjunction with acetonitrile–isopropanol reversed phase gradient, and a butanol extraction followed by water-methanol gradient on the same or a different reversed phase column. This covers the entire spectrum from very lipophilic lipid species (triglycerides, cholesterol-esters, etc.) to the relatively hydrophilic lipids (acyl-CoAs, LPAs, etc.). While this increases workload and commitment of mass spectrometry time, it is necessary when one's aim is to maximize coverage of the lipidome.

Continued developments in chromatography and mass spectrometry are driving ongoing improvements in analytical performance. Together with a well thought out sample preparation approach and optimized separation and mass spectrometer settings, we are guaranteed to detect and identify more lipid species. It will be important for the community to actively populate existing public databases with these findings. In short, we anticipate that the use of lipidomics will lead to exciting new discoveries in the field of lipid biology in the coming years.

## References and recommended reading

Papers of particular interest, published within the period of review, have been highlighted as:

• of special interest
•• of outstanding interest
